# Comparative analysis of mitochondrial genomes between the *hau* cytoplasmic male sterility (CMS) line and its iso-nuclear maintainer line in *Brassica juncea* to reveal the origin of the CMS-associated gene *orf288*

**DOI:** 10.1186/1471-2164-15-322

**Published:** 2014-04-30

**Authors:** Shuangping Heng, Chao Wei, Bing Jing, Zhengjie Wan, Jing Wen, Bin Yi, Chaozhi Ma, Jinxing Tu, Tingdong Fu, Jinxiong Shen

**Affiliations:** National Key Laboratory of Crop Genetic Improvement, National Center of Rapeseed Improvement in Wuhan, College of Plant Science and Technology, Huazhong Agricultural University, Wuhan, 430070 P.R. China; Key Laboratory of Horticulture Biology, Ministry of Education, College of Horticulture and Forestry Sciences, Huazhong Agricultural University, Wuhan, 430070 P.R. China; College of Agronomy, Northwest Agriculture & Forestry University, Yangling, Shaanxi 712100 P.R. China

**Keywords:** *Brassica juncea*, Mitochondrial, Cytoplasmic male sterility, *orf288*, Mitotype

## Abstract

**Background:**

Cytoplasmic male sterility (CMS) is not only important for exploiting heterosis in crop plants, but also as a model for investigating nuclear-cytoplasmic interaction. CMS may be caused by mutations, rearrangement or recombination in the mitochondrial genome. Understanding the mitochondrial genome is often the first and key step in unraveling the molecular and genetic basis of CMS in plants. Comparative analysis of the mitochondrial genome of the *hau* CMS line and its maintainer line in *B. juneca* (*Brassica juncea*) may help show the origin of the CMS-associated gene *orf288*.

**Results:**

Through next-generation sequencing, the *B. juncea hau* CMS mitochondrial genome was assembled into a single, circular-mapping molecule that is 247,903 bp in size and 45.08% in GC content. In addition to the CMS associated gene *orf288*, the genome contains 35 protein-encoding genes, 3 rRNAs, 25 tRNA genes and 29 ORFs of unknown function. The mitochondrial genome sizes of the maintainer line and another normal type line “J163-4” are both 219,863 bp and with GC content at 45.23%. The maintainer line has 36 genes with protein products, 3 rRNAs, 22 tRNA genes and 31 unidentified ORFs. Comparative analysis the mitochondrial genomes of the *hau* CMS line and its maintainer line allowed us to develop specific markers to separate the two lines at the seedling stage. We also confirmed that different mitotypes coexist substoichiometrically in *hau* CMS lines and its maintainer lines in *B. juncea*. The number of repeats larger than 100 bp in the *hau* CMS line (16 repeats) are nearly twice of those found in the maintainer line (9 repeats). Phylogenetic analysis of the CMS-associated gene *orf288* and four other homologous sequences in *Brassicaceae* show that *orf288* was clearly different from *orf263* in *Brassica tournefortii* despite of strong similarity.

**Conclusion:**

The *hau* CMS mitochondrial genome was highly rearranged when compared with its iso-nuclear maintainer line mitochondrial genome. This study may be useful for studying the mechanism of natural CMS in *B. juncea*, performing comparative analysis on sequenced mitochondrial genomes in *Brassicas*, and uncovering the origin of the *hau* CMS mitotype and structural and evolutionary differences between different mitotypes.

**Electronic supplementary material:**

The online version of this article (doi:10.1186/1471-2164-15-322) contains supplementary material, which is available to authorized users.

## Background

Cytoplasmic male sterility is a phenotypic trait that is widespread among plants and results in the inability of the plant to produce viable pollen [1]. Numerous studies have shown that cytoplasmic male sterility in plants is associated with aberrant recombination in the mitochondrial genome, which results in the production of chimeric ORFs that are expressed as novel polypeptides [2]. Since the first plant mitochondrial genome sequencing in *Arabidopsis*[3], a large number of mitochondrial genomes have been sequenced in angiosperm plants [4–10], especially in those that contain CMS cytoplasm. CMS-associated mitochondrial genome of crop species reported to date include *Beta vulgaris*[11], *Oryza sativa*[12–14], *Triticumae stivum*[15], *Zea mays*[16], *Brassica napus*[17, 18], *Raphanus sativus*[19, 20]. In this study, the mitochondrial genome of *hau* CMS line, its maintainer line and the normal type line “J163-4” were fully sequenced and assembled into a master circle. As in other higher plants, all three sequenced mitochondrial genomes had large sizes and distinctive features, including slow evolutionary rates, rapid rearrangement, frequent insertion, complex multipartite structures, specific modes of gene expression, cis- and trans-splicing, RNA editing and the use of universal genetic code [21]. Comparative analysis of the CMS line and its iso-nuclear maintainer line may help us verify the CMS-associated gene in *hau* CMS line, and contribute to a better understanding of the plant mitochondrial genome in *Brassicas*.

The male sterile *hau* CMS line (00-6-102A) emerged as a spontaneous male sterile mutant in *B. juncea*. The anthers in the *hau* CMS plants are replaced by thickened petal-like structures, and *hau* CMS sterility starts at the stamen primordium polarization stage, much earlier than the other four CMS systems used in *Brassicas* (*pol*, *ogu*, *nap*, and *tour*) [22]. A novel chimeric gene named *orf288* was found to be located downstream of the *atp6* gene and co-transcribed with this gene in the *hau* CMS sterile line. Subcellular localization analysis showed that this CMS-associated gene was translated in the mitochondria of male-sterile plants. Transgenic result also showed that ORF288 is associated with the male sterility of *hau* CMS in *Brassica juncea*[23].

In this study, we sequenced the complete mitochondrial genomes of *hau* CMS line, its iso-nuclear maintainer line and the normal type line “J163-4” in *B. juneca* using Roche/454 pyro-sequencing technology. Comparative analysis of the *hau* CMS mitochondrial genome further confirmed that *orf288* was a cytoplasmic male sterility-associated gene in *B. juneca*. The sequenced mitochondrial genomes may help us identify the mechanism of natural CMS and uncover the origin and structure of the *hau* CMS mitotype as well as understand evolutionary differences between the different mitotypes in *B. juncea*. Our data give new insight into the evolution of the *Brassicas* mitochondrial genome.

## Results

### The mitochondrial genomes of the *hau* CMS line and its maintainer line

**Figure 1:**
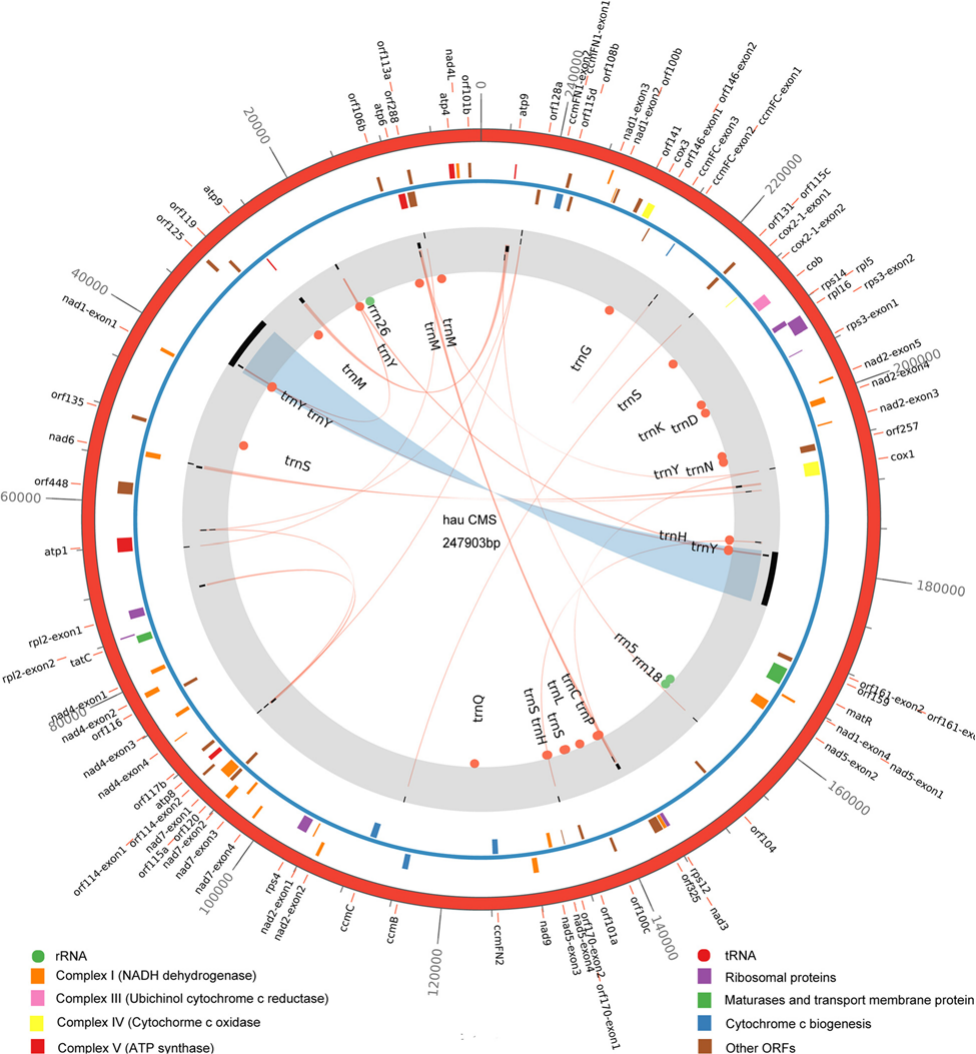
**A circular diagram of the** ***hau*** **CMS line mitochondrial genome.** From outside to inside: numbers on the outermost circle represent the physical map scaled in bp. Coding sequences transcribed in the clockwise and counterclockwise direction are drawn on the inside and outside of the second circle, respectively. Genes encoding proteins from the same complexes are similarly colored as are rRNAs and tRNAs in the inner circle. The third circle shows the locations of repeats larger than 100 bp with the most compelling evidence for recombination activity, and detailed information for repeats is shown in Additional file 5.

The mitochondrial genomes of the *hau* CMS line, its maintainer line and the normal type line “J163-4” were sequenced to an average depth of 52*, 196* and 69* multiple depths using Roche 454 FLX + pyro-sequencing technology. Sequences were assembled to 7, 3 and 4 contigs, respectively. The PCR primers used for the confirmation of contig linkage are listed in Additional file 1 and the mitochondrial DNA extracted from the etiolated seedlings in 7 days of the *hau* CMS line and its iso-nuclear maintainer line were used as templates. Results of the PCR amplification are presented in Additional file 2. A master circle was developed for each mitochondrial genome using a ‘parsimonious’ method [24]. Given that the mitochondrial genome of the *hau* CMS maintainer line and the line “J163-4” are identical to each other, we only performed comparative analysis of *hau* CMS line and its maintainer line in this study. Features of the *hau* CMS line and its maintainer line mitochondrial are shown in Table 1. The *B. juncea hau* CMS mitochondrial genome was assembled into a single, circular-mapping molecule with a size of 247,903 bp and GC content of 45.08%, both of which are close to the median values of other fully sequenced seed plant mitochondrial genomes. Coding sequences constitute approximately 20.52% of the genome, which contains 35 protein coding open reading frames (ORFs), 3 rRNA genes, 25 tRNA genes, and 29 unidentified ORFs (Figure 1). The size of its maintainer line is 219,863 bp, and the GC content is 45.23% with 23.43% of the sequence as coding sequence. The maintainer line mitochondrial genome contains 36 protein-coding genes, 3 rRNA genes, 22 tRNA genes, and 31 unidentified ORFs as shown in Additional file 3.

**Table 1:** **Summary features of mitochondrial genome contents in the** ***hau*** **CMS mitotype and its maintainer mitotype**

Feature	***hau*** CMS	Normal
Genome size (bp)	247,903	219,863
GC content (%)	45.08%	45.23%
Coding sequence (%)	20.52%	23.43%
Protein coding genes	35	36
ORFs	29	31
tRNA genes	25	22
rRNA genes	3	3

**Figure 2:**
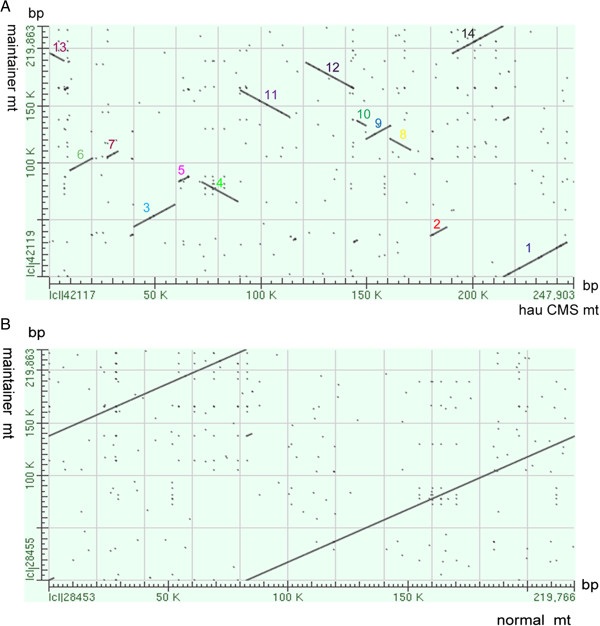
**Comparison syntenic region of** ***hau*** **CMS mitotype with its maintainer line mitotype and the normal. (A)** Comparisons between the *hau* CMS mitochondrial genome (horizontal axis) and its maintainer line mitochondrial genome (vertical axis) indicated that the nucleotide sequences of the syntenic region are well conserved; however, the syntenic order and direction were largely rearranged. The numbers refer to the syntenic regions between the *hau* CMS mitochondrial genome and its iso-nuclear maintainer line mitochondrial genome. **(B)** Alignment of the *hau* CMS maintainer line mitochondrial genome and the normal mitochondrial genome [GenBank: JF920288] sequenced by Chang in *Brassica juncea*. Apart from SNPs, they were consistent and no rearrangement was found between them.

After the initial sequence analysis, we compared the coding regions, ORFs of unknown function and repeats of the two circular mitochondrial genomes. The electron carrier complexes I, III, IV and V genes in the *hau* CMS line and its maintainer line were conserved, while the *rps7* gene encoding a subunit of ribosomal proteins in the *hau* CMS line was absent when compared with its maintainer line. Detailed information describing the tRNA gene content of the *hau* mitotype and its maintainer line (normal) mitotype is shown in Additional file 4. After a comparative analysis of the predicted ORFs in the two mitochondrial genomes, specific ORFs were found occurring in both mitochondrial genomes. We also plotted the syntenic regions using the bl2seq algorithm with the *hau* CMS line, its iso-nuclear maintainer line and the sequenced *B. juncea* [GenBank: JF920288] mitochondrial genome [18]. As shown in Figure 2A, the genomic arrangement of the *hau* CMS line mitochondrial genome was very divergent when compared with its maintainer line, with at least 14 apparent rearrangements. However, as shown in Figure 2B, when the *B. juncea* mitochondrial genome sequenced by Chang [18] was compared with the *hau* CMS maintainer line, no divergent genomic arrangement occurred except that of SNPs divergence. This result not only confirmed the accuracy of our sequence assembly but also showed that the *hau* CMS line mitochondrial genome was extensively rearranged when compared with its maintainer line.

### Repeats in the *hau* CMS line and its maintainer line

**Figure 3:**
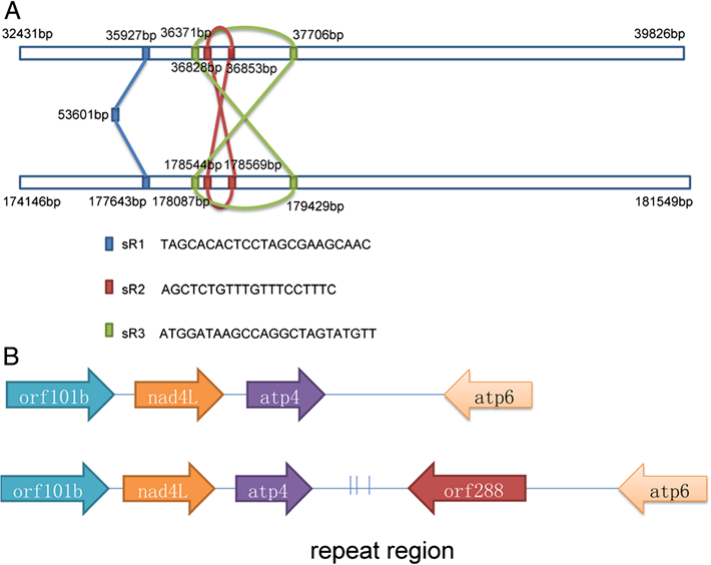
**The largest repetitive region and structural polymorphism surrounding** ***orf288*** **in the** ***hau*** **CMS mitochondrial genome. (A)** The largest repeats in the *hau* CMS mitochondrial genome are 7369 bp and 7404 bp, both of which showed 8 bp indel SNPs. There was also three small direct repeats named sR1, sR2 and sR3 in the largest repeats. A detailed nucleotide sequence of the three repeats is given above. **(B)** Three large repeats of more than 100 bp were located downstream of the CMS-associated *orf288* gene.

Repeats in the plant mitochondrial genome may be relevant to the rapid rearrangement, frequent insertion and complex multipartite structure in plant mitochondrial genomes. Based on mitochondrial genomes of the *hau* CMS line and its maintainer line, detailed information for repeated sequences greater than 100 bp was annotated in Additional file 5 and shown in Figure 1 and Additional file 3. The number of repeats greater than 100 bp in the *hau* CMS line (16 repeats) was almost two fold of that in its maintainer line (9 repeats). There were 8 direct repeats and 8 inverted repeats in the *hau* CMS line, as well as 5 direct and 4 inverted repeats in its maintainer line. The largest repeats in the *hau* CMS line were 7,396 bp and 7,404 bp with only 8 different indels between them (data not shown). The largest repeats in the maintainer line were 2293 bp, and both were direct repeats. Recombination across inverted repeats inverts the intervening sequences, whereas recombination across directly oriented repeats separates the genome into pairs of sub-genomic molecules [25]. Repeats smaller than 100 bp were also investigated in the mitochondrial genome of the *hau* CMS line and its maintainer line. Intriguingly, we observed many small repeats in the 2 sequenced mitochondrial genomes. In both the *hau* CMS line and its maintainer line, there were more than 200 short repeats smaller than 100 bp. Some large repeats also contained a few small repeats smaller than 100 bp. The largest repeat in the mitochondrial genome of the *hau* CMS line in Figure 3A was analyzed, which encompassed 5 small direct repeats. The border of this repeat, the initiation codon of these small repeats and the nucleotide sequences of these three small repeats were marked. The same phenomenon was also found in other large repeats in the *hau* CMS line and its maintainer line mitochondrial genomes. Apparently, these small repeats were part of the large repeats. However the relations between these large and small repeats remain unclear. It is known that sub-genomic molecules are produced from the MC molecule through intra-molecular recombination. Different sub-genomic molecules may constitute different mitochondrial genomes through recombination of these direct and inverted repeats. The short repeated sequences in higher plant mitochondria are usually inactive and may play key role in irreversible recombination producing a new stable mitochondrial genome structure [26]. These short repeats as part of the larger repeats in mitochondrial genome may help us to answer how the large repeats emerged, expanded and then gave rise to complex multipartite structures of plant mitochondrial genomes. Three large repeats larger than 100 bp were also found between syntenic regions of *atp4* and *atp6* (Figure 3B). They were located downstream of *orf288*, which may be relevant to the emergence of this CMS-associated gene.

### The specific ORFs of different mitochondrial genomes

**Figure 4:**
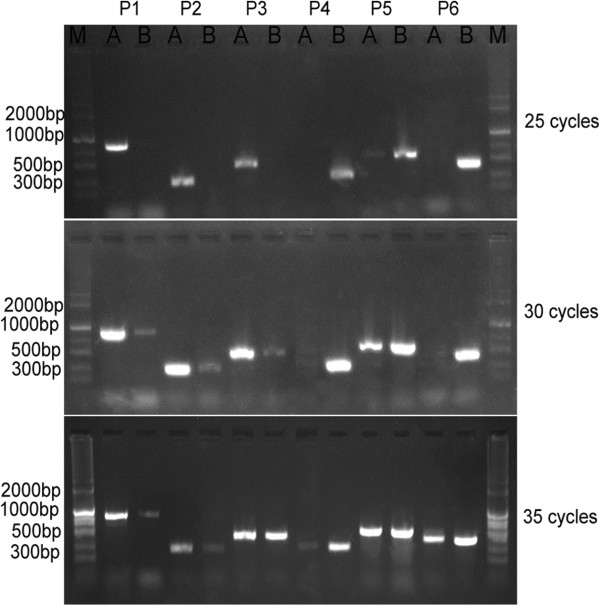
**PCR amplification in the mitochondrial genomes of the** ***hau*** **CMS line and its maintainer.** The *hau* CMS mitotype and the normal mitotype coexisted in both the *hau* CMS line 00-6-102A **(A)** and its maintainer line 00-6-102B **(B)**, but were substoichiometrically different in these cytotypes. Different PCR cycles (25, 30, 35) were used to amplify these different open reading frames. Primer pairs P1, P2 and P3 targeted the *hau* CMS mitotype-specific sequences *orf288*, *orf325* and *orf170*, respectively, whereas P4, P5 and P6 targeted its maintainer line (normal) mitotype-specific sequences. All PCR reactions used 50 ng mtDNA as template. The PCR assay indicated that the *hau* CMS mitotype and normal mitotypes coexisted in *Brassica juncea*, but the content was substoichiometrically different form the two cytoplasm types. M: DNA ladder.

We further analyzed those ORFs of unknown function in the sequenced mitochondrial genomes in this study. There were 29 and 31 such ORFs in the *hau* CMS line and its maintainer line, respectively. By virtue of mitochondrial rearrangement and indel mutations, specific ORFs were found in the *hau* CMS line and its maintainer line mitochondrial genome. There were 5 mitotype specific ORFs in the *hau* CMS line and its maintainer line separately. Sequence analysis revealed that *orf113a* in the *hau* CMS line had only SNPs differences when compared with *orf113b* in its maintainer line. Although *orf117b* in the *hau* CMS line had not been detected in its maintainer line, it was detected in the sequenced mitochondrial genomes reported by Chang [18]. Thus, we chose only 3 ORFs from each of the 2 lines. Among them *orf288*, *orf325* and *orf170* were *hau* mitotype-specific ORFs, while *orf109*, *orf293* and *rps7* were the maintainer line specific ORFs (Table 2). PCR amplification of the mitochondrial specific SCAR markers was developed based on these specific ORFs from the two sequenced mitochondrial genomes. They were both efficient in distinguishing the *hau* CMS line from its maintainer line at the seedling stage. We confirmed these by PCR analysis after developing the mitochondrial specific ORF markers which are shown in Additional file 1. As demonstrated in Figure 4, the P1, P2 and P3 primers combinations were specific to the A line (*hau* specific mitotype) while P4, P5 and P6 primers were specific to the B line (its iso-nuclear maintainer line) after 25 cycles. However, after 30 and 35 cycles, *hau* CMS specific ORFs were amplified slightly in the maintainer line, and the *hau* CMS maintainer line specific ORFs were also amplified in the *hau* CMS line, especially with the P3, P5 and P6 primers. It is noteworthy that there was evidence suggesting that substoichiometrically different mitotypes coexisted in *hau* CMS lines and their maintainer lines as reported by Chen [9].

**Table 2:** **Mitotype-specific ORFs and protein coding genes between the** ***hau*** **CMS line and its maintainer line**

Mitotype	ORF	Similarity of predicted protein	Location
*hau* mitotype specific	*orf325*	*nad3* and *rps12* genes, partial sequence [*Brassica napus*]	Between syntenic regions 12 and 10
	*orf170*	ADN44176.1 photosystem 1 subunit A, partial (chloroplast)	In the syntenic region 12
	*orf288*	CAA58667.1 *orf263* (mitochondrion) [*Brassica tournefortii*]	Between syntenic regions 6 and 13
Normal mitotype specific	*orf109*	YP_717154.1 hypothetical protein BrnapMp057 [*Brassica napus*]	Between syntenic regions 5 and 6
	*orf293*	YP_004927826.1 *orf293* (mitochondrion) [*Brassica rapa* subsp. *campestris*]	The edge of syntenic region 12
	*rps7*	NP_085579.1 ribosomal protein S7 [*Arabidopsis thaliana*]	Between syntenic regions 6 and 7

**Figure 5:**
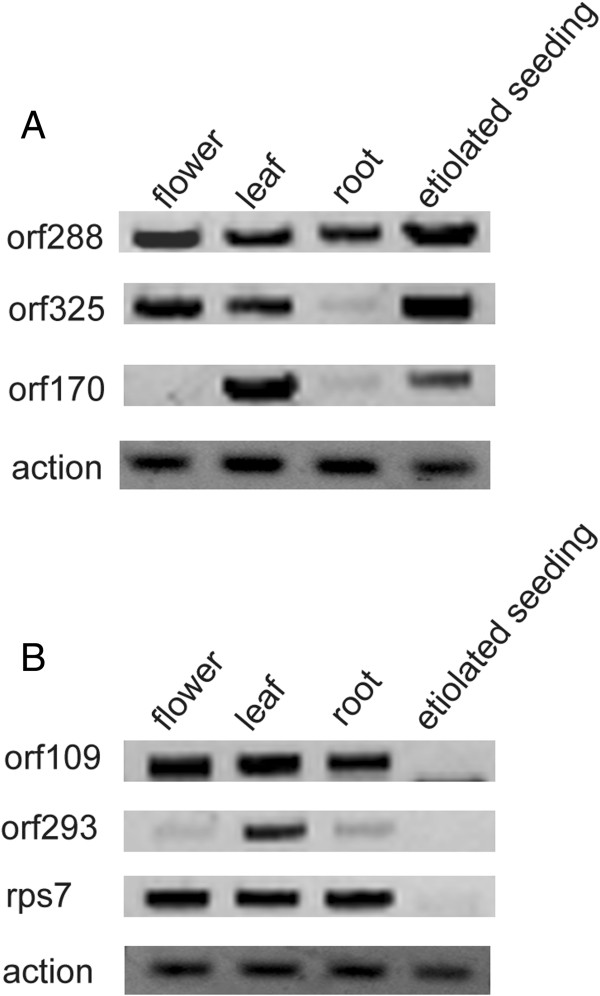
**Expression of the unknown function ORFs in the** ***hau*** **CMS line and its maintainer line. (A)** The expression of the *hau* CMS mitochondrial genome specific *orf288*, *orf325* and *orf170* in four different tissues. The CMS-associated gene *orf288* is expressed in the flower, leaf, root and etiolated seedings of *B. juncea* and is carried by the *hau* CMS mitochondrial genome. However, *orf325* did not appear in the root and *orf170* only appeared in the leaf and etiolated seedings 7 days after germination. **(B)** *orf109* and *rps7* both appeared in the flower, leaf, and root. However, *orf293*, which is similar to *orf288*, only appeared in the leaf of *hau* CMS maintainer line.

Subsequently, total RNA was isolated from flower buds, fresh leaves, roots and hypocotyls (etiolated seedlings). We examined the transcript pattern of these specific ORFs in the *hau* CMS line and its maintainer line. As shown in Figure 5, *orf288* was constitutively expressed in all tissues tested, while *orf325* was not detectable in the roots and *orf170* only appeared in leaves and the etiolated seedlings of the *hau* CMS line. In its maintainer line, *orf293* was only expressed in the leaves, while the *orf109* and *rps7* were undetectable at the etiolated seedling stage. Apart from *orf288*, which was known to be the CMS-associated gene in the *hau* CMS line, understanding the function of these ORFs requires further investigation.

### The *hau* CMS-associated gene *orf288* in *Brassica juncea*

**Figure 6:**
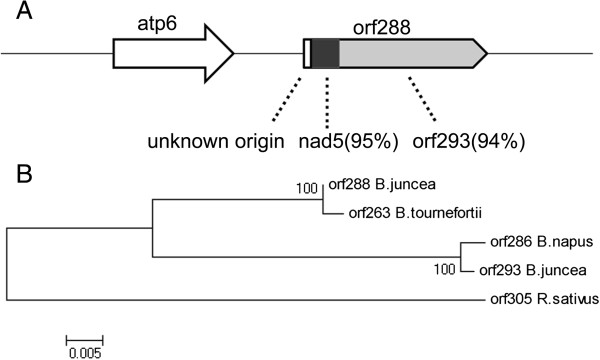
**The structure of** ***orf288*** **and phylogenetic tree based on conserved** ***orf288*** **genes in mitochondrial genomes. (A)** The *hau* CMS-associated gene *orf288* was located downstream of *atp6*. As for *orf263* in *Brassica tournefortii* mitochondrial genome, the nucleotide identities (%) of the mitochondrial genome-derived fragments to the chimeric gene *orf288* were shown. **(B)** Phylogenetic analysis of the CMS-associated gene *orf288* in *Brassicaceae. Orf263* was associated with cytoplasmic male sterility in *Brassica tournefortii*, Multiple sequence alignment used to construct the phylogenetic trees *orf286*, *orf293* and *orf305* shows they were separated in terms of sequence homology from the mitochondrial genomes of *B. napus*, *B. juncea* and *R. sativus*. Bootstrap values are shown at the nodes and the bar indicates the rate of nucleotide substitution per site.

CMS is often associated with specific open reading frames (ORFs) in plant mitochondrial genomes, but the origin of many CMS-associated genes and the mechanism of this phenomenon are still unclear. Utilizing next-generation sequencing, many more plant mitochondrial genomes have been released, especially for CMS-associated genomes of various crops such as rice, maize, wheat, sorghum and rapeseed (*B. napus*). Value of mitochondrial genome information is also well established in the study of evolutionary patterns and processes of CMS-associated genes in plants. Results from transgenics showed that *orf288* was responsible for the male sterility of *hau* CMS in *B. juncea*[23]. CMS-associated genes in different mitochondrial genomes are often located downstream of the genes encoding components of the electron transport respiratory chain. For example, *orf224* in the *B.napus polima* CMS line, *orf79* in the rice BORO CMS line, and *orf138* in the *ogura* CMS line are all located downstream of *atp6*. The *orf522* in the sunflower PET1 CMS line is located downstream of *atp1*[2]. Almost all these CMS-associated genes were chimeric genes and had transmembrane domains. The transmembrane domains of these specific open reading frames in *hau* CMS line were predicted using TMHMM server version 2.0. This revealed that apart from *orf288*, *orf325* also had the transmembrane domains, albeit its function is still unknown. The *hau* CMS-associated gene *orf288* was also located downstream of *atp6*, but how the CMS-associated gene emerged remained unclear. The 3 large repeats located downstream of *orf288* might have been relevant to the emergence of the CMS-associated gene. Like most CMS genes, *orf288* has similarities to known functional mitochondrial genes. Apart from the unknown origin (1–24 bp), the chimeric *orf288* gene also contains a 94 bp partial sequence of *nad5*, a subunit of complex I in the electron transport chain system. This is similar to the case of the 5′ region of the gene *orf263* as reported in alloplasmic male sterile *Brassica tournefortii*[27]. The 3′ region was also similar to another predicted mitochondrial ORF, *orf293* in *B. juncea* (Figure 6A). This result suggested that this recombinant structure evolved recently. By way of BLAST alignment analysis, it can be seen that *orf263*, *orf286*, *orf293* and *orf305* were homology sequenced separately from the sequenced mitochondrial genomes of *B. tournefortii, B. napus*, *B. juncea* and *R. sativus* in *Brassicaceae*. Other than *orf263*, the CMS-associated gene in *Brassica tournefortii* and *orf305* was previously reported by Christopher A. Makaroff in a cytoplasmic male sterility radish [28]. The other two open reading frames only appeared in the male fertility mitochondrial genomes of *B. napus* and *B. juncea*. A maximum-likelihood inference-based dendrogram was used to generate phylogenetic trees based on conserved *orf288* genes in mitochondrial genomes (Figure 6B). Molecular phylogenetic analysis indicated that *orf288* bore a strong similarity to *orf263* in *Brassica tournefortii* and the mitochondrial genomes of *B. napus* and *B. juncea* might be closer to each other than *R. sativus*.

**Figure 7:**
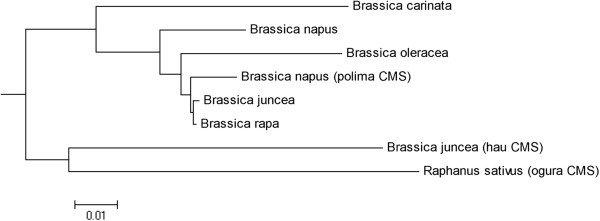
**A phylogenetic tree relating the eight mitochondrial genomes sequenced in** ***Brassicas*** **.** The *hau* CMS mitotype and *ogura* CMS mitotype were both diverged from the other six sequenced mitochondrial genomes in *Brassicas*. The bar indicates the rate of nucleotide substitution per site.

We also comparatively analyzed the mitochondrial genome of *hau* CMS with the mitochondrial genomes of *nap* [AP006444], *cam* [JF920285], *jun* [JF920288], *ole* [JF920286], *car* [JF920287], *polima* [FR715249] and *ogura* [AB694744] in *Brassicas*[17–20]. A phylogenetic guide tree (Figure 7) and locally collinear blocks (Additional file 6) relating these eight sequenced mitochondrial genomes were calculated by using Neighbor Joining of the Mauve alignment system. The 141.8 kb segment from 173,638 bp to 315,446 bp in *ole* mitochondrial genome was deleted for the reason that Tandem repeats >10 kb in total length without an anchor are ignored by MAUVE. Cluster analysis of the eight mitochondrial genomes showed that the mitochondrial genomes of *hau* CMS and *ogura* CMS are both diverged from the other six mitochondrial genomes in *Brassicas*. And the evolutionary relationships among the six analyzed homologous mitotypes are consistent with previous research by Chang [18]. These results confirmed the *hau* CMS mitotype which caused male sterility in *Brassicas* might be heterogeneous when compared with the other related mitotypes at molecular level.

## Discussion

### Comparative analysis of the mitochondrial genome of the *hau* CMS line with its maintainer line

Over the past two decades, our knowledge of the organization and evolution of mitochondrial genomes has been rapidly expanding as a large number of fully sequenced mitochondrial genomes have been published along with their structure, expression and evolutionary profiles. In light of the pivotal role of the CMS line in crop breeding and its convenience for studying the cytoplasmic and nuclear interaction, we compared the mitochondrial genomes of the *hau* CMS line and its iso-nuclear maintainer line to investigate the origin of the *hau* CMS mitotype and to dissect the structural and evolutionary differences between the different mitotypes. Mitochondrial genome of the *hau* CMS line (247,903 bp) was larger than its maintainer line (219,863 bp) and the repeats (>100 bp) appeared noticeably more frequent than those in its maintainer line. The size of plant mitochondrial genome was relevant to the repeats it contained, and the repeats in the mitochondrial genomes also revealed the structural dynamics of the mitochondrial genome in plant development via intramolecular and intermolecular recombination. In this study, we report that small repeats (<50 bp) might contribute to larger repeats (>100 bp) in the sequenced *hau* CMS mitochondrial genome, but the emergence of these small repeats and their function in the *hau* CMS line and its maintainer line mitochondrial genomes requires further study.

As reported in rice [13], maize [29], pearl millet [30], wheat [31], *B. napus*[17] and cybrids in *Brassicaceae*[32], our results also confirmed that substoichiometrically different mitotypes coexist in mitochondrial genomes of the *hau* CMS line and its maintainer line in *B. juncea*. The ratio of the alternative genomes may be variable, but in plants, the usually prevalent main genome is accompanied by sublimons—substoichiometric mitochondrial DNA (mtDNA) molecules [33]. In plants, the relative copy number of recombination-derived sub-genomic DNA molecules within mitochondria is controlled by nuclear genes and a genomic shifting process can result in their differential copy number suppression to nearly undetectable levels [34]. Most of the mitotype-specific ORFs in one mitochondrial genome present at a substoichiometric level in the other genome. At 25 cycles of PCR amplification, only templates carrying specific ORFs were able to produce a detectable PCR product in the *hau* CMS line and its maintainer line, and when the cycles were up to 30 to 35, all primer pairs (P1-P6) used were able to amplify specific ORFs in *hau* CMS line and its maintainer line, but the pattern of amplification differed in the 2 lines. The substoichiometric amount of specific ORFs from one genome to the other is thus sufficient to be detected by PCR amplification but not enough to have been picked up by the coverage depth of the 454 sequencing that was performed [13]. Although substoichiometrically different mitotypes coexist in mitochondrial genomes of different plants, the molecular mechanism of such coexistence in different mitotypes requires further study. The coexistence of different mitotypes may play a prominent role in the coordination of nuclear and mitochondrial interaction and also make valuable contributions to the hybrid vigor in different crop plants.

Heterosis plays an irreplaceable role in China’s high-yield crop production, and male sterility, which is a prerequisite for the mass production of hybrid seeds, acts as a key factor [35]. Cytoplasmic male sterility (CMS) in plant, which is determined by the mitochondrial genome is associated with a pollen sterility phenotype and caused by mitochondrial genome mutation. Identification of a CMS-associated gene and uncovering the mechanism of this trait may facilitate plant breeding. With the sequenced mitochondrial genome, more novel mitochondrial genome types and molecular markers for cytoplasm classifications will be identified. Recently, Chang et al. [18] sequenced the mitotypes of *cam* (*B. rape*), *ole* (*B. oleracea*), *jun* (*B. juncea*) and *car* (*B. carinata*) and analyzed them together with previously sequenced mitotypes of *B. napus* (*pol* and *nap*) to show the evolutionary mechanism of mitochondrial genome formation in *Brassica*. Molecular markers such as RFLPs, AFLPs, SCARs, and SSRs were used to distinguish the CMS line from its maintainer line in the *Brassicas*. Based on the sequenced mitochondrial genome, specific SCAR markers (the *hau* CMS line specific primers combination P1, P2 and P3 and its iso-nuclear maintainer line specific primers combination P4, P5 and P6) were also developed to separate the *hau* CMS line from its maintainer line at the seedling stage.

### The origin and emergence of the CMS-associated gene *orf288* in *B. juncea*

A large number of CMS-associated genes have been found in crop species, but the origin and precise mechanism of CMS remains elusive. In different CMS systems, the CMS-associated genes show little or no structural relationship. Often the CMS-associated genes or loci are located close to an atp gene or contain parts of a gene encoding an ATPase subunit and are co-transcribed with flanking mt-genes [36]. Although the CMS phenotype also occurs at different stages during reproductive development, they were the root cause of male sterility. In our study, the CMS-associated gene *orf288* located downstream of *atp6* and is co-transcribed. The transgenic result further verified that *orf288* is associated with the male sterility of *hau* CMS in *Brassica juncea*[23]. As a chimeric gene, *orf288* is composed partially of *nad5* and *orf293* in *B. juncea*, and there were 3 large repeats larger than 100 bp located downstream of *orf288*. These repeats may be related to the formation of the CMS-associated gene. Although it has relatively high similarity with *orf263* in alloplasmic male sterile *Brassica tournefortii* at the nucleotide level, the restorer lines for *tour* CMS systems were found to be ineffective for restoring fertility in the *hau* CMS line. This may suggest that they were different from each other [22]. A comparative analysis of the mitochondrial genome of the *hau* CMS line and its maintainer line further confirmed that *orf288* was CMS-associated gene in *hau* CMS line in *B. juncea*.

Voluminous evidence suggests that mitochondrial gene expression can affect the function of the nuclear gene products that control floral development. In Honglian cytoplasmic male sterile rice, the CMS-associated gene *orfH79* impaired mitochondrial function via interaction with *P61* (a subunit of electron transport chain complex III), and resulted in an energy production dysfunction and oxidative stress in mitochondria, which may work as retrograde signals leading to abnormal pollen development [37]. In Wild Abortive CMS rice, WA352 accumulates preferentially in the tapetum of anthers, where it inhibits COX11 function in peroxide metabolism and triggers premature tapetal programmed cell death and consequent pollen abortion. These CMS models provided a mechanistic link between the gain of function of a newly identified mitochondrial CMS gene product and the loss of activity of the essential nuclear-encoded mitochondrial protein through their detrimental interaction [38]. It is thus likely that different recombinations in different plant mitochondrials gave rise to different chimeric genes that caused male sterility through interaction with genes in the anther development pathways and eventually caused male sterility in different CMS systems. This hypothesis might explain why dissimilar CMS genes in different plants all caused similar phenotypic male sterility. The nature of different CMS-associated genes that interact with the anther development pathways still needs to be studied further.

## Conclusions

The *hau* CMS mitochondrial genome was highly rearranged as was reported for mitochondrial genomes in CMS lines of other crops. The chimeric CMS-associated gene *orf288* was composed of 94 bp partial sequences of *nad5* (a subunit of complex I in the electron transport respiratory chain system) and 749 bp sequences that were highly similar to *orf293* in its maintainer line. Three large repeats downstream of *orf288* may be related to the formation of the CMS-associated gene in the *hau* CMS line. These findings may help us to identify the mechanism of natural CMS in *B. juncea* and to uncover the origin of the *hau* CMS mitotype and the structural and evolutionary differences between different mitotypes.

## Methods

### Plant materials

The *hau* CMS line (00-6-102A) used in this study was originally discovered as a spontaneous male-sterile mutant in *B. juncea* in the experimental field at Huazhong Agricultural University in 1999. The maintainer line (00-6-102B) was iso-nuclear to the *hau* CMS line in *B. juncea*. A cultivar trilocular line, J163-4, in *B. juncea* with fertility anther was also used as control in our study [39]. The anthers in the *hau* CMS plants are replaced by thickened petal-like structures and the sterility of the *hau* CMS initiates at the stamen primordium polarization stage [22]. Seeds from the *hau* CMS line, its iso–nuclear maintainer line and the normal type line “J163-4” were harvested in an experimental field at Huazhong Agricultural University in 2011.

### Isolation of mitochondrial DNA and total RNA

Discontinuous Percoll gradient centrifugation was used to separate highly purified mitochondria from 7-day-old etiolated seedlings (Additional file 7) for *hau* CMS line, its maintainer line and the normal type line “J163-4” from *B. juncea*. A 100 g sample of each seedling-stage hypocotyls from the 3 different lines were homogenized in 200 ml homogenization medium (0.4 M mannitol, 5 mM EDTA, 8 mM cysteine, 10 mM tricine, 1% BSA, 1% polyvinyl-pyrrolidone, pH 7.8). The homogenate was filtered using four pieces of Miracloth and centrifuged at 1000 g for 5 minutes. The resulting supernatants were centrifuged at 18,000 g for 15 minutes and the pellet was re-suspended in wash buffer (0.4 M mannitol, 1 mM EDTA, 10 mM MOPS-KOH and 1% BSA) to repeat the above procedure at 1000 g for 5 minutes and 18,000 g for 15 minutes. The reaction was terminated with the addition of 20 mM EDTA. The pellet was resuspended in wash buffer and layered onto a step gradient consisting of 15%, 20%, 28% and 40% Percoll in 0.4 mM mannitol, 1% BSA and 10 mM MOPS-KOH. Purified mitochondrial were removed from the 20% and 28% interphase (Additional file 7). The pellet was resuspended in wash buffer without EDTA and 25 mg/ml DNase (Roche 104159) was added at room temperature for 1-3 h. Finally, samples were centrifuged at 18,000 g for 20 minutes and resuspended in the lysis buffer (50 mM Tris–HCl, 10 mM EDTA, 1% SDS, and 200 mg/ml proteinase K (Sigma) at room temperature for 3 h [40]. The CTAB method was used to obtain the purified mitochondrial DNA [41]. Total RNA was isolated from flower buds, fresh leaves, roots, and hypocotyls using Trizol (Invitrogen) according to the manufacturer’s protocol.

### The sequencing strategy

The complete mitochondrial genomes of the *hau* CMS line, its maintainer line and the normal type line in *B. juneca* were sequenced using the Roche 454 FLX + pyro-sequencing technology. The sequencing service was provided by Personal Biotechnology (Shanghai, China). Nucleotide sequences of 13,130,330 bp, 43,164,917 bp and 15,240,642 bp in total were obtained from the 00-6-102A, 00-6-102B and J163-4 lines, respectively. The average sequence depth was 52, 196 and 69. The sequence was assembled to 7, 3 and 4 contigs in the *hau* CMS line, its maintainer lines and the normal type line “J163-4”. The genomic PCR products sequence between contigs was obtained by Sanger sequencing.

### Sequence analysis

ORF Finder, BLASTX, BLASTN, and tRNA-SE were used to identify mitochondrial genes, rRNA, and tRNA. Artemis software [42], which allowed the use of a threshold to identify ORFs, was used to identify ORFs whose function was unknown. Both of these mitochondrial genome sequences were assembled using the Seqman software (DNAStar). Bl2seq (http://www.ncbi.nlm.nih.gov/) was used to do sequence alignment to find the syntenic region in the sequenced mitochondrial genomes. ClustalW2 and MEGA 4 were used for a phylogenetic analysis of CMS associated gene *orf288* in *Brassicaceae*. Circos was used to visualize data and information of the mitochondrial genome of the *hau* CMS line and its maintainer line [43]. Progressive Mauve was used for multiple alignment among the 8 sequenced mitochondrial genomes in *Brassicas*[44]. The mitochondrial genome sequences of the *hau* CMS line and its maintainer line have been deposited to the GenBank Database under accession numbers: KF736092 and KF736093.

## Electronic supplementary material

Additional file 1: **Primers used in this study.** (XLSX 16 KB)

Additional file 2: **Validation of contig linkage through PCR analysis.** Line number refers to the primer combinations used for validating the gap between the contigs in the *hau* CMS mitotype and the normal mitotype in Additional file 1. PCR confirmed the bridge sequences of the gaps in the *hau* CMS line and its maintainer line mitochondrial genome in A and B, respectively. (TIFF 419 KB)

Additional file 3: **A circular diagram of the** ***hau*** **CMS maintainer line mitochondrial genome in** ***B. juneca.*** As for the *hau* CMS mitochondrial genome diagram shown in Figure 1, numbers on the outermost circle represent the physical map scaled in kb. Coding sequences transcribed in the clockwise and counterclockwise directions are drawn on the inside and outside of the second circle, respectively. Genes coding proteins from the same complexes are similarly colored as are rRNAs and tRNAs in the inner circle. The third circle shows the locations of repeats larger than 100 bp with the most compelling evidence for recombination activity, and detailed information for repeats is shown in Additional file 5. (TIFF 858 KB)

Additional file 4: **tRNA content of the** ***hau*** **CMS mitotype and its iso-nuclear maintainer mitotype.** (XLSX 10 KB)

Additional file 5: **Repeated sequences (>100 bp, 99% homology) in the** ***hau*** **CMS mitotype and its iso-nuclear maintainer mitoype.** (XLSX 15 KB)

Additional file 6: **Locally collinear blocks identified among the eight sequenced mitochondrial genomes in** ***Brassicas.*** Mauve visualization of locally collinear blocks identified among the eight sequenced mitochondrial genomes in *Brassicas*. Each contiguously colored region is a locally collinear block (LCB) region without rearrangement of homologous backbone sequence. LCBs below a genome’s center line are in the reverse complement orientation relative to the reference genome. Lines between genomes trace each orthologous LCB through every genome. Large gray regions within an LCB signify the presence of lineage-specific sequence at that site. (TIFF 3 MB)

Additional file 7: **Extraction of high-quality mitochondrial genome DNA from** ***B. juncea.*** Seedlings etiolated for 7 days were used in the isolation of mitochondrial. Percoll differential centrifugation and density gradient centrifugation were used to separate the purified mitochondrial DNA. (TIFF 2 MB)
