# Supplementary figures and images for: Comparative analysis of mitochondrial genomes between the hau cytoplasmic male sterility (CMS) line and its iso-nuclear maintainer line in Brassica juncea to reveal the origin of the CMS-associated gene orf288
Source: BMC Genomics. 2014 Apr 30;15(1):322. doi: 10.1186/1471-2164-15-322 (PMC4035054; doi:10.1186/1471-2164-15-322)

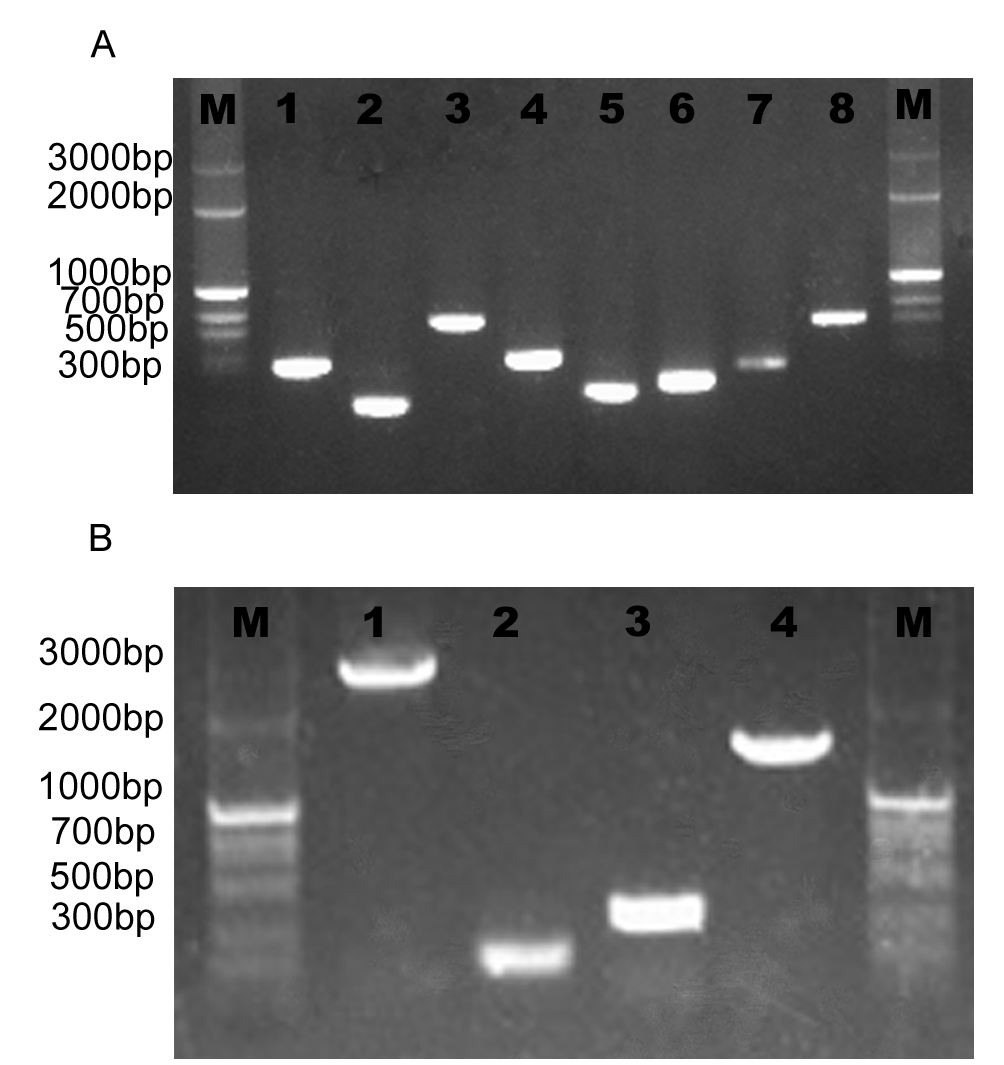

Supplement: Supplementary file 2 — Additional file 2: Validation of contig linkage through PCR analysis. Line number refers to the primer combinations used for validating the gap between the contigs in the hau CMS mitotype and the normal mitotype in Additional file 1. PCR confirmed the bridge sequences of the gaps in the hau CMS line and its maintainer line mitochondrial genome in A and B, respectively. (TIFF 419 KB) [file 12864_2013_6022_MOESM2_ESM.tiff]

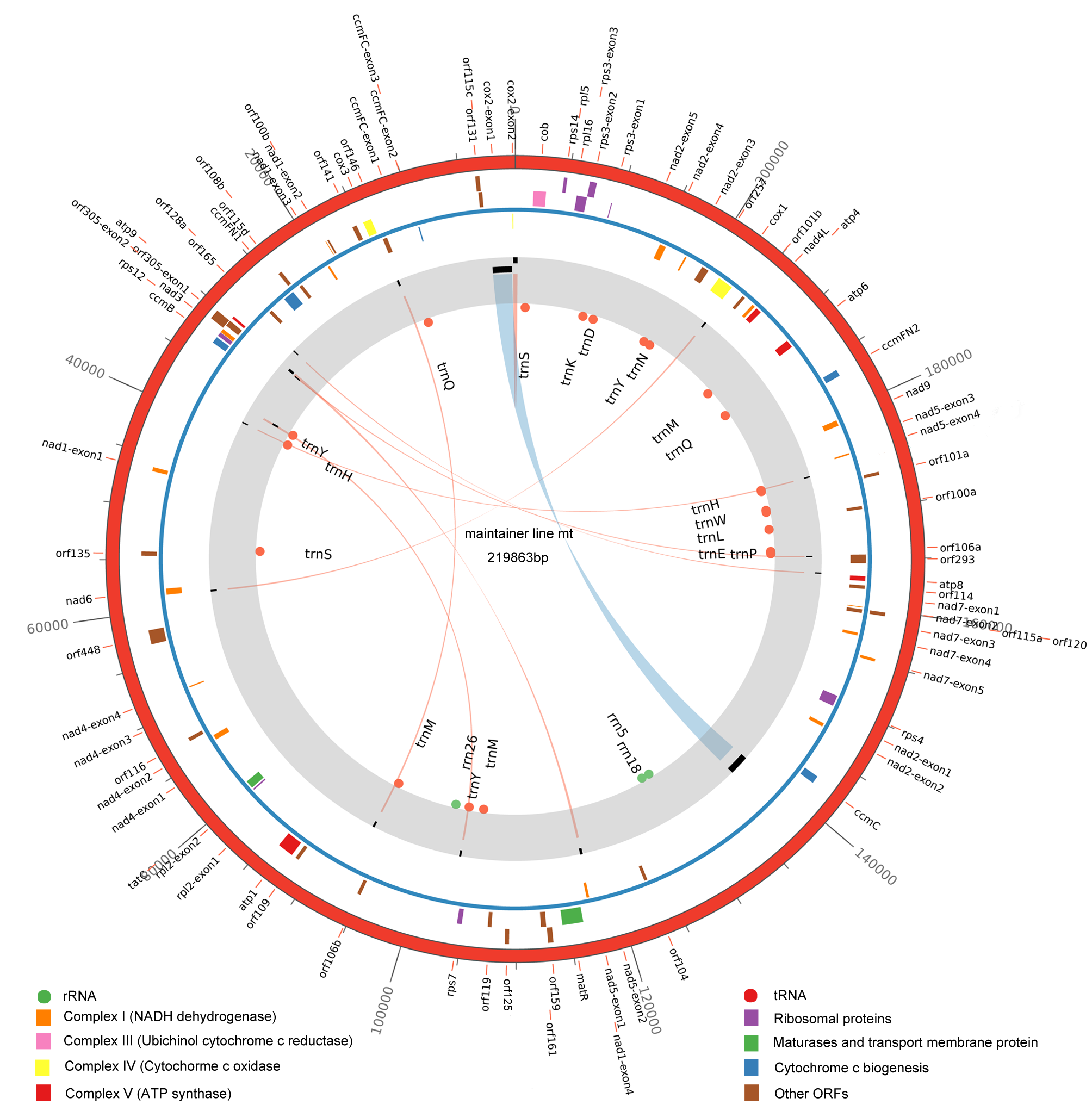

Supplement: Supplementary file 3 — Additional file 3: A circular diagram of the hau CMS maintainer line mitochondrial genome in B. juneca. As for the hau CMS mitochondrial genome diagram shown in Figure 1, numbers on the outermost circle represent the physical map scaled in kb. Coding sequences transcribed in the clockwise and counterclockwise directions are drawn on the inside and outside of the second circle, respectively. Genes coding proteins from the same complexes are similarly colored as are rRNAs and tRNAs in the inner circle. The third circle shows the locations of repeats larger than 100 bp with the most compelling evidence for recombination activity, and detailed information for repeats is shown in Additional file 5. (TIFF 858 KB) [file 12864_2013_6022_MOESM3_ESM.tiff]

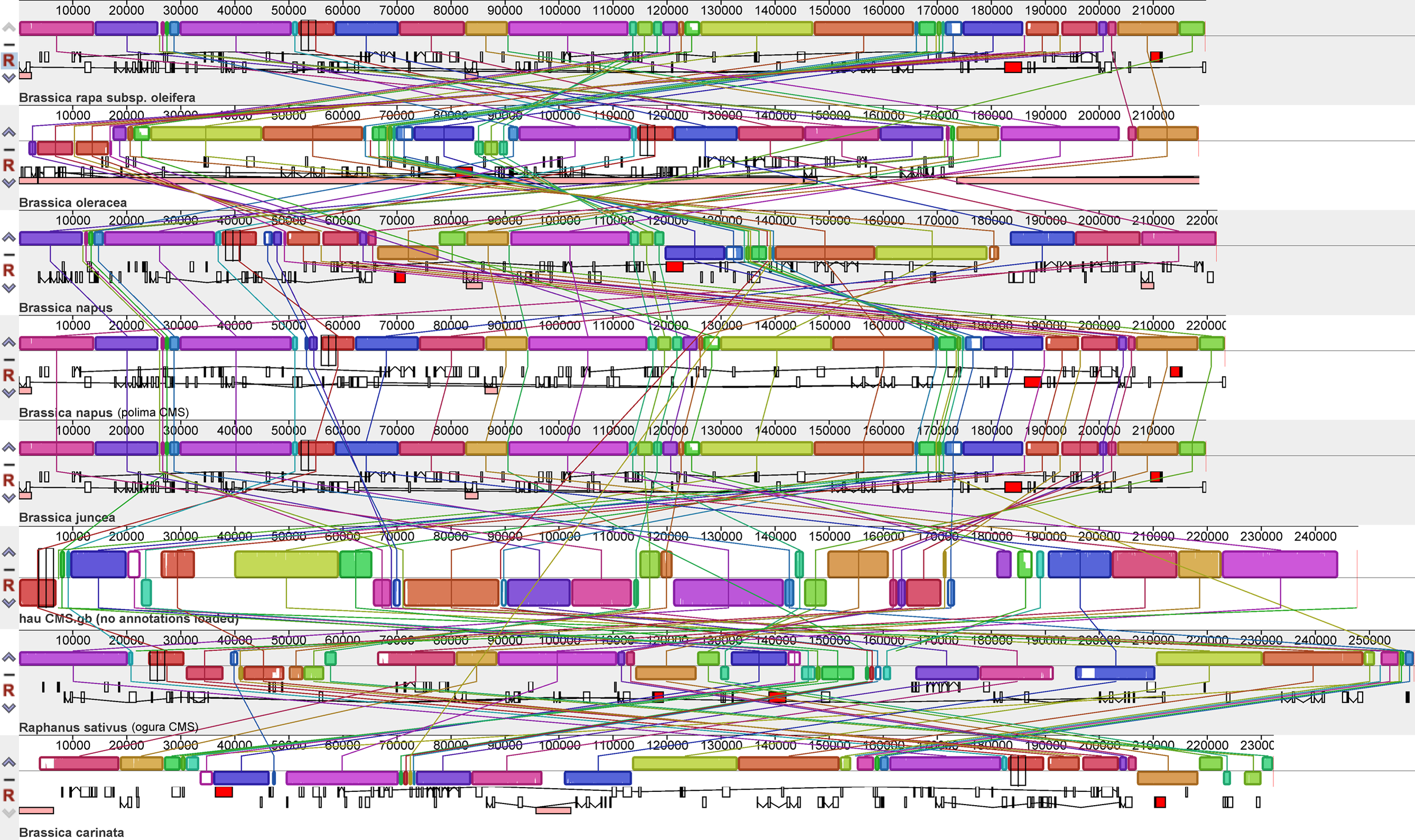

Supplement: Supplementary file 6 — Additional file 6: Locally collinear blocks identified among the eight sequenced mitochondrial genomes in Brassicas. Mauve visualization of locally collinear blocks identified among the eight sequenced mitochondrial genomes in Brassicas. Each contiguously colored region is a locally collinear block (LCB) region without rearrangement of homologous backbone sequence. LCBs below a genome’s center line are in the reverse complement orientation relative to the reference genome. Lines between genomes trace each orthologous LCB through every genome. Large gray regions within an LCB signify the presence of lineage-specific sequence at that site. (TIFF 3 MB) [file 12864_2013_6022_MOESM6_ESM.tiff]

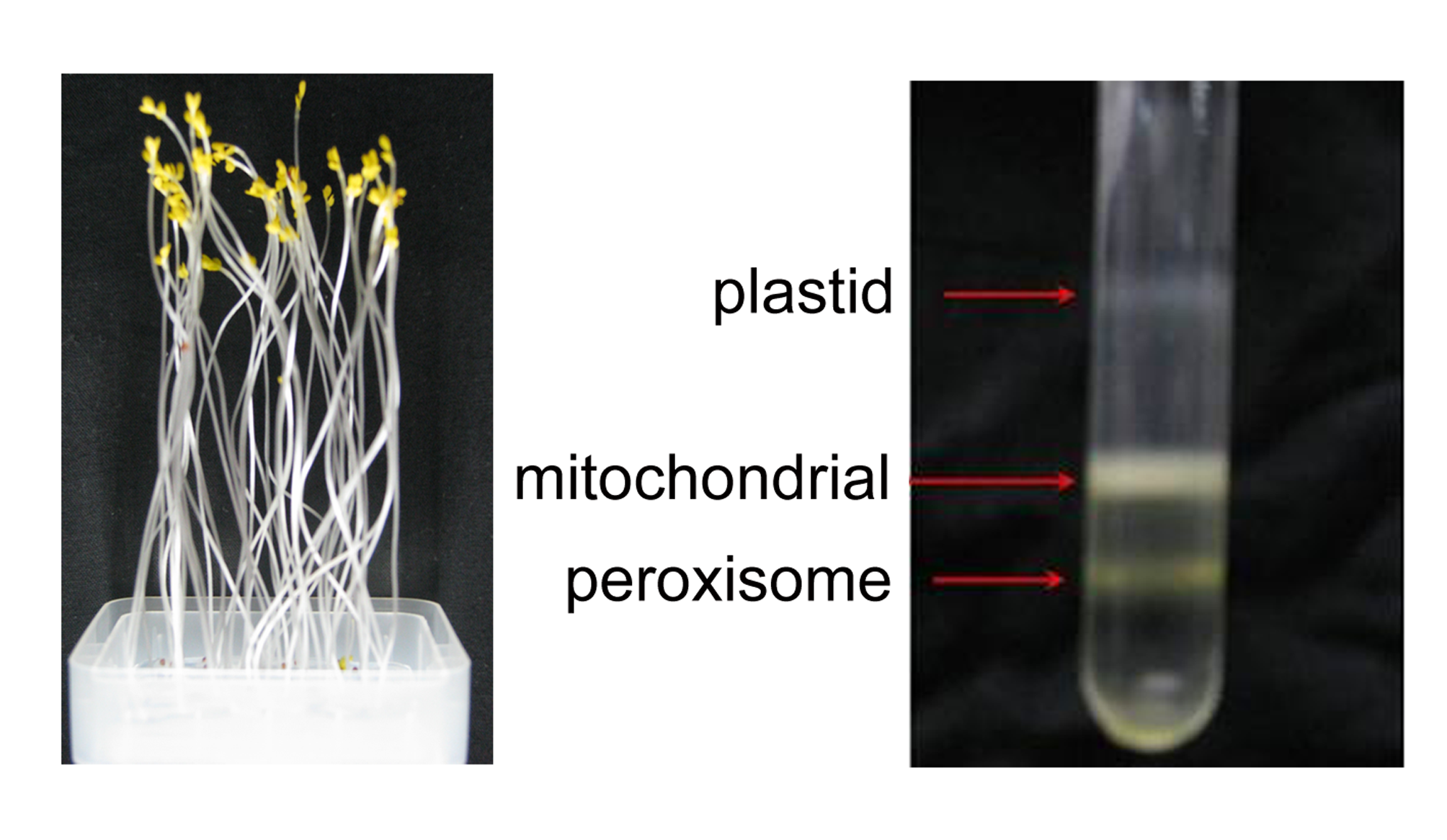

Supplement: Supplementary file 7 — Additional file 7: Extraction of high-quality mitochondrial genome DNA from B. juncea. Seedlings etiolated for 7 days were used in the isolation of mitochondrial. Percoll differential centrifugation and density gradient centrifugation were used to separate the purified mitochondrial DNA. (TIFF 2 MB) [file 12864_2013_6022_MOESM7_ESM.tiff]
